# Cognitive and motor dual task gait training exerted specific training effects on dual task gait performance in individuals with Parkinson’s disease: A randomized controlled pilot study

**DOI:** 10.1371/journal.pone.0218180

**Published:** 2019-06-20

**Authors:** Yea-Ru Yang, Shih-Jung Cheng, Yu-Ju Lee, Yan-Ci Liu, Ray-Yau Wang

**Affiliations:** 1 Department of Physical Therapy and Assistive Technology, National Yang-Ming University, Taipei, Taiwan; 2 Department of Neurology, Mackay Memorial Hospital, Taipei, Taiwan; 3 Department of Rehabilitation Medicine, Hualien Tzu Chi Hospital, Hualien, Taiwan; University of California Irvine, UNITED STATES

## Abstract

Gait impairments in Parkinson’s disease (PD) are aggravated under dual task conditions. Providing effective training to enhance different dual task gait performance is important for PD rehabilitation. This pilot study aimed to investigate the effects of cognitive and motor dual task gait training on dual task gait performance in PD. Eighteen PD participants (n = 6 per training group) were assigned to cognitive dual task gait training (CDTT), motor dual task gait training (MDTT), or general gait training (control) group randomly. The training was 30 min each session, 3 sessions per week for 4 weeks. Primary outcomes including gait performance during cognitive dual task, motor dual task, and single walking were assessed at pre- and post-training. The results showed decreased double support time during cognitive dual task walking after CDTT (-17.1±10.3%) was significantly more than MDTT (6.3±25.6%, p = .006) and control training (-5.6±7.8%, p = .041). Stride time variability during motor dual task walking decreased more after MDTT (-16.3±32.3%) than CDTT (38.6±24.0%, p = .015) and control training (36.8±36.4%, p = .041). CDTT also improved motor dual task walking performance especially on gait speed (13.8±10.71%, p = .046) stride length (10.5±6.6%, p = .046), and double support time (-8.0±2.0%, p = .028). CDTT improved single walking performance as well on gait speed (11.4±5.5%, p = .046), stride length (9.2±4.6%, p = .028), and double support time (-8.1±3.0%, p = .028). In summary, our preliminary data showed 12-session of CDTT decreased double support time during cognitive dual task walking, and MDTT reduced gait variability during motor dual task walking. Different training strategy can be adopted for possibly different training effects in people with PD.

## Introduction

Parkinson’s disease (PD) is a neurological degenerative disease which leads to motor impairments such as functional walking. Dual task walking is one of the functional walking that is essential for daily life. In daily living, it often requires walking while performing simultaneous cognitive or motor task, such as talking with a friend (cognitive dual task walking) or carrying a cup of coffee (motor dual task walking). It has been reported that gait impairments in people with PD are particularly noticeable under dual task conditions included decreased gait speed and stride length and increased stride-to-stride variability[1–4].

In the early stages of motor skill acquisition, the cortical regions of the brain play a major role in movement regulation, as movements become learned and automatic which are thought to be controlled by the basal ganglia[5]. In people with PD, learned movements such as walking can still be generated when attention is focused on the performance. However, during dual task walking, the attention may need to engage in performing the secondary task, leaving responsibility for regulating the more automatic walking task to the defective basal ganglia circuitry. Therefore, gait speed decreased and gait automaticity as indicated by the stride variabilities increased in people with PD during dual task walking as compared to their usual walking [4]. The interference of secondary task during walking may lead to risks of falls during different dual task walking [2].

It is noted that dual task performance can be improved by learning in younger and older adults, and in people with PD. According to motor learning studies not only intensity but also exact practice conditions are critical in practice-related improvement.[6] Yogev-Seligmann et al. demonstrated cognitive dual task gait training improved cognitive dual task gait speed and gait variability in their single PD group study [7]. Strouwen et al. reported whether gait and cognitive tasks trained consecutively or concurrently led to similar improvements in dual-task gait velocity according to their study on PD population [8]. On the other hand, based on the principles of task-specific training, we have previously demonstrated that gait training with cognitive task improved cognitive dual task gait performance and gait training with motor task improved motor dual task gait performance in people with chronic stroke [9]. Therefore, different types of dual task gait training exert benefit to improve different dual task walking performance in people with stroke [9]. Whether training specificity is significant in people with PD is not known. However, such information may provide effective training protocol to enhance different dual task gait performance for people with PD. Thus, the purpose of present study was to evaluate the effects of cognitive and motor dual task gait training on dual task gait performance in individuals with PD. We hypothesized that both cognitive and motor dual task training improved dual task gait performance, and different dual task training may exert different effect on dual task gait performance in people with PD.

## Methods

### Participants

Participants who were diagnosed as idiopathic PD by neurologist were recruited from medical centers in Taipei, Taiwan. The diagnostic criteria for PD were based on presenting bradykinesia with resting tremor and/or rigidity [10]. The inclusion criteria included: (i) Hoehn and Yahr stages from I to III, (ii) walking independently, and (iii) the mini-mental state examination (MMSE) score is >24. The exclusion criteria included: (i) unstable medical condition, (ii) motor fluctuations or severe dyskinesia which might interfere the training, and (iii) any history of other diseases known to interfere with participation in the study. This study procedures were approved by the Institutional Review Boards of Mackay Memorial Hospital and National Yang-Ming University. The registration of the trial was not done right after the IRB approval because the authors were less aware of the required prospective registration. However, this trial was registered in Thai Clinical Trials Registry (TCTR20180801001) on 27^th^ July 2018.

### Experimental design

**Fig 1:**
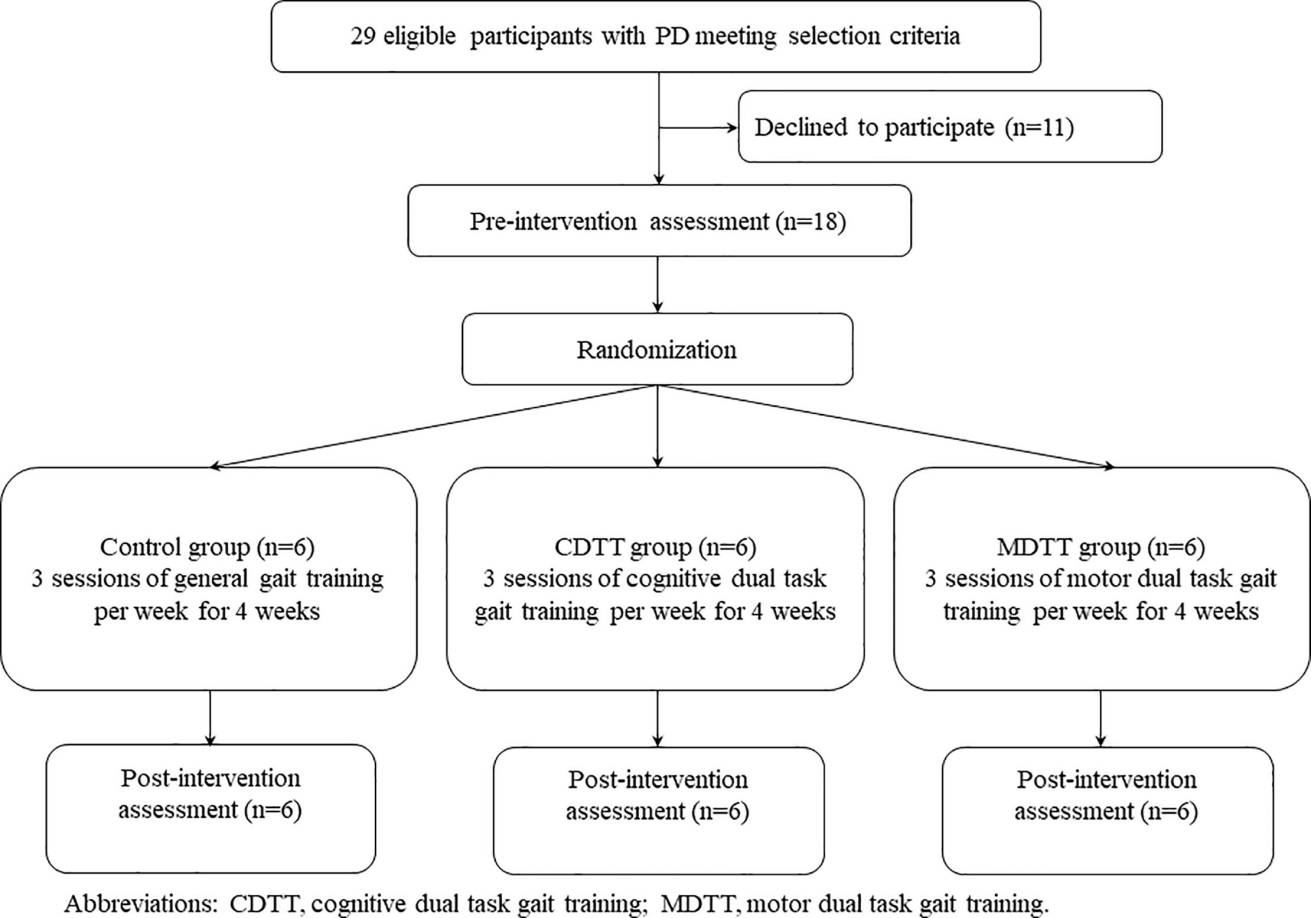
Study flowchart.

This study was a single-blinded parallel randomized controlled trial. An individual who was not involved with the study selected the sealed envelope to assign participants to one of the three treatment groups: cognitive dual task gait training (CDTT), motor dual task gait training (MDTT), or control group by block randomization (allocation ratio = 1). The training session was 30 minutes, 3 sessions per week, for a total of 4 weeks with 12 sessions. All the training sessions were in charged by the same well-trained physical therapist. All outcomes were measured by the same assessor who was blinded to group assignment. The assessment was taken place on the day before and after the intervention program. (Fig 1) The measurements and intervention were conducted with patients in the “on” state.

### Interventions

The Template for Intervention Description and Replication (TIDieR) checklist was used to describe the intervention. The intervention was administered by the same physical therapist on one-on-one basis in exercise laboratory of university. Participants in the CDTT group were instructed to execute cognitive tasks while diverse walking conditions. The cognitive dual tasks included: (1) walking while repeating words, (2) walking while counting a 3-digit number forward, (3) walking while counting a 3-digit number backward, (4) walking while answering simple question “yes” or “no”, (5) walking while reciting a shopping list, (6) walking while talking, (7) walking while reciting a short sentence backward, and (8) walking while singing. Walking tasks included walking forward, obstacle crossing walking, walking on an S-shaped route, tandem walking, and walking backward. Participants were progressively trained with increased difficulty of tasks [9]. Training progression is shown in S1 Table.

Participants in the MDTT group were instructed to perform motor tasks during different walking conditions on a level surface. The motor dual tasks included: (1) walking while holding one ball (diameter = 20 cm) with both hands; (2) walking while bouncing a basketball (diameter = 24.6 cm) with both hands; (3) walking while bouncing a basketball (diameter = 24.6 cm) with either hand; and (4) walking while bouncing one basketball (diameter = 24.6 cm) with one hand and concurrently holding another basketball with the other hand (diameter = 20 cm). Walking tasks included walking forward, obstacle crossing walking, walking on an S-shaped route, tandem walking, and walking backward. Participants were progressively trained with increased difficulty of tasks [9] Training progression is shown in S2 Table.

Participants in CDTT group and MDTT group were asked to concentrate on “both” tasks as much as possible during training sessions.

Participants in the control group received general gait training on level surface for 15 min, including walking forward, walking on an S-shaped route, walking and obstacle crossing, tandem walking, and walking backward each session followed by 15 min of treadmill training at comfortable speed. Training progression is shown in S3 Table.

### Primary outcomes

The gait performance was the primary outcome in this study. Gait parameters were recorded during following conditions: (1) single task walking with self-selected speed, (2) walking with serial subtracting by three, starting from a randomized 3-digit number (e.g. 100, 97, 94…) (cognitive dual task walking), and (3) walking while carrying a tray with a bottle of water in front of the subject with both hands (motor dual task walking). To avoid the effects of practice or fatigue, the three walking conditions were performed in random order and each condition was measured 3 times with a 60 second rest in between. The average value of three repeats was used for data analysis.

Gait parameters were recorded by a GAITRite system with 4.30 m long and 0.61 m wide pressure-sensitive area. When subject walks along the walkway, the contact time and location of each footfall are recorded and analyzed on a laptop using Microsoft Excel 2013 to calculate temporal-spatial parameters of walking. Gait parameters were recorded including speed, cadence, stride length, double support time, and stride time variability. Dual task cost of gait speed (DTC-speed) were determined the dual task interference [11–13]. The formula was shown as following.

DTC-speed = (dual task walking speed–single task walking speed)/single task walking speed * 100% [14].

### Secondary outcomes

The secondary outcomes included timed up and go test (TUG), freezing of gait (FOG), and fall efficacy scale-international (FES-I).

The functional mobility and balance were determined by TUG test in the present study [15]. It is a simple test that has been used in clinics and researches to present a general ability to safely move around. In this test, participants are instructed to stand up from a chair, walk 3 meters, turn around, walk back to the chair, then sit down. The time needed to complete this task was recorded. A high reliability of this test has been documented in patients with PD [16].

Present study used the freezing of gait questionnaire (FOGQ) which was the only validated tool to subjectively assess FOG. It assesses the frequency of FOG, disturbances in gait, and the clinical features conceptually associated with gait and motor function. The score ranges from 0 to 24, and the higher scores represent more severity of FOG. Furthermore, FOGQ has been demonstrated a good test-retest reliability [17].

The FES-I is widely used in elderly persons to indicate concerns about falling. There are 16 items assessing functional tasks and social-related activities, and scoring ranges from 1 to 4. A higher score indicates a greater concern about falling. The validity of the FES-I has been reported for older adults [18, 19].

### Statistical analysis

Descriptive statistics were generated for all variables, and distributions of variables were expressed as median (95% confidence interval). The Kruskal-Wallis one-way ANOVA by ranks and chi-square test were used for basic data analysis. The Wilcoxon signed ranks test was used for within group comparisons. The Kruskal-Wallis one-way ANOVA by ranks was used for the score changes in between-group comparisons with Mann-Whitney U test for post hoc test. All the statistical tests were not corrected for multiple testing. The statistical significance was set at p < .05.

The sample size in the present study was 18 after power calculation utilizing G*Power 3.1, based on one of the primary outcomes effect (dual task gait performance) [9]. Using an effect size of 0.8, a type I error of 0.05, and a 70% power, at least 6 patients in each group were required for identifying statistically significant differences in dual task walking performance (one-way ANOVA).

## Results

There were 29 individuals identified as potential subjects. Of these, 18 participants provided written informed consent for participating this study and were randomly assigned to the CDTT, MDTT, or control group (n = 6 for each group) (Fig 1). There was no significant baseline difference among groups (Table 1), and no change in type or dosage of medication during the study period in every participant. Similarly, there was no significant differences among groups in all outcome measures at the pre-intervention assessment. In addition, no adverse effects such as falls were reported during the training periods.

**Table 1:** Demographic characteristics of included participants.

	**CDTT (n = 6)**	**MDTT (n = 6)**	**Control (n = 6)**	**p-value**
Age (years)	65.0 (57.5–75.8)	69.5 (65.0–77.0)	66.5 (55.5–76.5)	0.50
Gender (male/female)	4/2	4/2	4/2	1.00
Disease duration (years)	5.5 (2.8–10.5)	5.0 (0.1–12.5)	3.0(0.3–10.0)	0.76
Hoehr and Yahr stage	2.0 (1.6–2.6)	2.0 (1.8–2.7)	1.5 (0.9–2.4)	0.41
MMSE	27.0 (25.9–28.1)	27.5 (25.1–28.9)	28.0 (27.1–28.9)	0.28
Levodopa (mg/day)	892.0 (432.2–1307.5)	798.0 (534.8–1074.2)	557 (205.7–1234.7)	0.76

Data are presented as mean±SD or frequency

Abbreviations: CDTT, cognitive dual task gait training group; MDTT, motor dual task gait training group; MMSE, mini-mental state examination

Table 2 shows the gait performance during serial subtraction tasking (cognitive dual task walking) at pre- and post-intervention for three groups. In the CDTT group, stride length increased significantly by 19.0% (5.1%-29.9%, p = 0.031) and double support time decreased significantly by 19.8% (-27.9%—6.4%, p = 0.031) compared to pre-training (Table 2). Furthermore, the decrease in double support time was significantly more in the CDTT group as compared with the MDTT and control group (p = 0.026, p = 0.041 respectively).

**Table 2:** The pre, post, and changes of cognitive dual task walking performance of participants in different groups.

	**CDTT (n = 6)**		**MDTT (n = 6)**		**Control (n = 6)**		**p-value**
	Pre	Post	Pre	Post	Pre	Post	
Speed (cm)	59.8 (41.3–93.5)	74.5 (56.6–970)	72.1 (35.9–96.3)	56.9 (33.8–76.3)	60.2 (36.6–80.6)	60.6 (46.0–74.6)	.097
Changes (%)		13.1 (-8.3–47.2)		-21.0 (-78.4–87.3)		5.1 (-14.9–31.5)	
Stride length (cm/s)	86.8 (63.1–107.6)	99.7 (78.0–120.1)^#^	97.8 (47.7–114.4)	82.6 (59.4–101.4)	79.9 (61.7–92.0)	82.7 (75.7–89.4)	.526
Changes (%)		19.0 (5.1–29.9)		-2.5 (-24.1–42.0)		4.6 (-7.4–27.0)	
Cadence (steps/min)	90.3 (70.2–119.4)	92.1 (69.5–120.3)	98.4 (72.2–121.5)	91.0 (53.3–114.0)	95.0 (68.2–110.1)	95.1 (69.2–106.8)	.797
Changes (%)		1.0 (-15.0–17.2)		-4.8 (-55.1–42.8)		-2.6 (-10.7–9.3)	
Double support time (%)	36.5 (29.1–44.0)	30.5 (22.7–37.9)^#^	33.8 (24.2–50.0)	38.6 (29.6–53.4)	39.1 (31.6–49.3)	35.4 (31.2–44.4)	.012*
Changes (%)		-19.8 (-27.9- -6.4) ^a^^b^		15.6 (-10.6–43.1)		-3.6 (-13.8–2.7)	
Stride time variability (%)	6.1 (3.2–10.5)	5.3 (2.6–9.9)	6.5 (2.4–11.7)	6.2 (0.4–17.7)	8.5 (3.0–17.8)	7.4 (0.69–25.4)	.755
Changes (%)		-19.5 (-73.4–99.7)		-4.1 (-60.6–133.3)		14.3 (-36.4–80.3)	
Dual task cost-speed (%)	27.6 (14.8–45.1)	25.3 (18.6–35.8)	22.3 (10.2–40.0)	20.6 (9.3–53.4)	31.9 (11.8–58.7)	26.3 (12.3–50.9)	.850
Changes (%)		-3.0 (-45.5–36.0)		-45.5 (-132.6–38.9)		11.0 (-34.4–21.1)	

Data are presented as median (95% confidence interval)

Abbreviations: CDTT, cognitive dual task gait training group; MDTT, motor dual task gait training group

#, p <0.05 for within group comparisons

*, p <0.05 for between group comparison

^a^, p <0.05 as compared with CDDT

^b^, p <0.05 as compared with control group

Regarding motor dual task gait performance (walking while carrying a tray), results showed significant improvements in double support time (-8.2%, -10.5- -5.9%, p = 0.031) were also noted in CDTT group compared with pre-training measurements (Table 3). However, such improvement in CDTT group did not reach to a statistical significance as compared with MDTT and control group. The stride time variability was decreased only after MDTT (19.3%, -50.2–17.5%), and such change was significantly different from that of the CDTT and control group (p = 0.015 and p = 0.041 respectively).

**Table 3:** The pre, post, and changes of motor dual task walking performance of participants in different groups.

	**CDTT (n = 6)**		**MDTT (n = 6)**		**Control (n = 6)**		**p-value**
	Pre	Post	Pre	Post	Pre	Post	
Speed (cm/s)	83.1 (67.0–102.3)	91.7 (74.0–118.2)	82.8 (44.8–114.3)	72.2, 58.0–87.5)	82.5 (61.2–91.8)	82.1 (67.3–90.8)	.343
Changes (%)		17.0 (2.5–25.0)		-0.7 (-43.2–57.8)		0.8 (-21.1–34.4)	
Stride length (cm)	96.4 (80.3–106.5)	103.7 (90.6–114.8)	91.9 (51.1–114.5)	88.4 (66.5–103.5)	85.7 (68.4–96.4)	87.0 (73.2–100.1)	.623
Changes (%)		11.1 (3.5–17.4)		6.0 (-22.5–46.7)		2.0 (-14.9–28.6)	
Cadence (steps/min)	107.8 (92.2–126.3)	110.7 (93.4–131.5)	120.9 (93.0–134.2)	106.7 (94.3–113.3)	114.1 (102.3–121.5)	107.9 (99.2–122.2)	.163
Changes (%)		3.5 (-0.2–7.9)		-9.1 (-26.5–24.0)		-2.5 (-7.4–5.5)	
Double support time (%)	31.5 (27.4–34.9)	28.9 (25.6–31.7)^#^	34.7 (24.7–44.9)	32.4 (29.8–37.3)	31.9 (27.5–41.5)	32.0 (29.9–36.1)	.526
Changes (%)		-8.2 (-10.0- -5.9)		-4.1 (-25.9–29.2)		2.9 (-13.0–7.2)	
Stride time variability (%)	2.9 (2.1–3.8)	3.8 (2.6–5.5)^#^	4.7 (3.4–7.3)	4.5 (2.9–5.4)	3.4 (2.6–4.7)	4.5 (3.0–6.9)	.026*
Changes (%)		43.9 (13.4–63.8)		-19.3 (-50.2–17.5) ^a^^b^		36.7 (-1.3–75.0)	
Dual task cost-speed (%)	11.0 (5.2–14.9)	8.6 (2.5–14.3)	9.9 (-6.1–22.1)	7.5 (2.1–15.0)	19.6 (6.5–26.2)	12.9 (2.4–21.0)	.645
Changes (%)		4.4, (-195.6–130.5)		42.5 (-38.0–156.9)		17.5 (-1645.9–761.3)	

Data are presented as median (95% confidence interval)

Abbreviations: CDTT, cognitive dual task gait training group; MDTT, motor dual task gait training group

#, p <0.05 for within group comparisons

*, p <0.05 for between group comparison

^a^, p <0.05 as compared with CDDT

^b^, p <0.05 as compared with control group

The results of gait performance during single walking after training are shown in Table 4. After CDTT, the increases in speed by 12.9% (5.6–17.2%, p = 0.031) and stride length by 9.3% (4.4–14.0%, p = 0.031), and decrease in double support time by 7.6% (4.9–11.2%, p = 0.031) were significant compared to pre-training measurements. However, the changes after CDTT did not differ significantly from the changes after MDTT or control training.

**Table 4:** The pre, post, and changes of regular walking performance of participants in different groups.

	**CDTT (n = 6)**		**MDTT (n = 6)**		**Control (n = 6)**		**p-value**
	Pre	Post	Pre	Post	Pre	Post	
Speed (cm/s)	91.9 (73.3–115.5)	103.7 (85.2–123.8)^#^	91.2 (55.2–113.5)	80.5 (64.5–94.5)	93.4 (71.0–113.3)	88.9 (73.8–106.5)	.064
Changes (%)		12.9 (5.6–17.2)		-8.0 (-35.2–42.3)		-3.3 (-14.0–12.8)	
Stride length (cm)	105.6 (87.7–117.3)	113.6 (98.3–124.7)^#^	103.5 (67.4–120.7)	93.6 (74.3–109.1)	98.0 (78.6–111.2)	102.1 (86.7–108.6)	.238
Changes (%)		9.3 (4.4–14.0)		-3.4 (-17.1–19.2)		-0.6 (-10.0–18.6)	
Cadence (steps/min)	111.4 (94.5–127.5)	111.3 (96.4–129.0)	113.4 (90.2–123.7)	110.5 (95.3–114.5)	116.3 (105.3–127.2)	108.7 (97.5–125.0)	.136
Changes (%)		1.7 (-0.5–3.7)		-4.2 (-19.0–19.0)		-5.0 (-8.9–0.0)	
Double support time (%)	28.9 (24.8–32.4)	26.1 (22.6–30.0)^#^	30.3 (24.0–37.8)	31.2 (28.3–34.5)	29.4 (25.1–36.5)	29.9 (26.9–33.4)	.056
Changes (%)		-7.6, (-11.2- -4.9)		2.1 (-10.9–18.9)		1.4 (-11.1–9.3)	
Stride time variability (%)	2.6 (1.4–4.1)	3.3 (2.2–4.5)	4.9 (3.5–5.7)	4.0 (3.2–5.2)	4.5 (3.3–5.2)	4.7 (3.0–5.9)	.074
Changes (%)		13.7 (-24.6–87.7)		-9.5 (-22.6–6.2)		19.8 (-28.6–46.9)	

Data are presented as median (95% confidence interval)

Data are presented as mean±SD

Abbreviations: CDTT, cognitive dual task gait training group; MDTT, motor dual task gait training group

#, p <0.05 for within group comparisons

The time needed to complete the TUG test decreased significantly at the post-training in the CDTT group (p = 0.046). The FOGQ score decreased significantly at the post-training (p = 0.042) in the MDTT group. However, these changes did not reach to a group significant difference (Table 5).

**Table 5:** The pre, post, and changes of secondary outcome measures of participants in different groups.

	**CDTT (n = 6)**		**MDTT (n = 6)**		**Control (n = 6)**		**p-value**
	Pre	Post	Pre	Post	Pre	Post	
Timed up and go test (s)	9.9 (8.2–11.9)	8.7 (7.5–9.8)^#^	12.2 (6.6–19.1)	9.3 (7.8–11.9)	9.8 (5.0–16.4)	10.1 (6.9–11.5)	.502
Changes (%)		-12.8 (-24.2- -1.7)		-13.4 (-39.5–9.1)		-2.8 (-27.2–13.0)	
Freezing of gait questionnaire	8.5 (2.3–11.7)	5.5 (0.9–11.1)	13.0 (2.3–19.4)	10.5 (1.5–16.1)^#^	4.5 (0.0–9.3)	2.5 (0.4–5.3)	.767
Changes (%)		-25.0 (-72.1–19.6)		-19.1 (-38.5- -1.5)		-6.3 (-96.8–174.1)	
Fall efficacy scale-international	34.5 (20.1–49.2)	32.0 (18.9–45.8)	36.0 (23.0–57.4)	35.5 (22.9–49.8)	26.0 (16.6–40.1)	26 (17.6–38.1)	.330
Changes (%)		-4.6 (-12.2–3.8)		-5.8 (-22.0–7.9)		-1.9 (-7.8–6.3)	

Data are presented as mean±SD

Abbreviations: CDTT, cognitive dual task gait training group; MDTT, motor dual task gait training group

#, p <0.05 for within group comparison

## Discussion

This pilot randomized controlled trial is the first study to compare the effects of different types of dual task gait training on dual task gait performance in individuals with PD. In the present study, we found CDTT can be more effective to decrease double support time during cognitive dual task walking than MDTT and control exercise. However, the MDTT was more effective in reducing the gait variability than the CDTT and the control exercise in people with PD. Previously, we have demonstrated significant training-specific effects of different dual task gait training in stroke patients [9]. Such training-specific effects is also noted to certain degree in people with PD. In addition, the cognitive dual task training can also improve the motor dual task walking and single walking performance in our participants.

Brauer et al. found that the cognitive dual task walking speed and stride length were improved after a 20-min cognitive dual task gait training, but the cadence and double support time were not improved in PD patients [20]. Yogev-Seligmann et al. indicated the improvement of speed and stride time variability in the trained and untrained cognitive dual task walking performance after 12 sessions of cognitive dual task gait training, suggesting that transfer of training might be possible in people with PD [7]. In the present study, the measurement for dual task gait performance was different from the dual task gait training program, and we also found both the stride length and double support time improved after cognitive dual task gait training. The decrease in double support time, which may indicate a better balance control during dual task walking [21], was more significant than the motor dual task training or the general gait training. Although the averaged cognitive dual task walking speed did not change significantly after cognitive dual task gait training, there was an increase by 9.4 cm/s after training which was greater than minimal clinically important difference for gait speed (5 cm/s). Therefore, the transfer effect to untrained cognitive dual task gait performance was also supported in the present study.

In the present study, we further noted that the cognitive dual task gait training improved gait speed, stride length, and double support time during motor dual task walking and single walking, which could not be achieved by motor dual task gait training or general gait training. Previous study suggested that difficulties with dual tasking are likely to be exacerbated by nonmotor symptoms, most notably by cognitive dysfunction in PD patients [8]. In the pre-intervention assessment of present study, participants (n = 6 per training group) showed more decrease in gait speed and cadence, and increase in double support time and stride time variability during cognitive dual task walking than motor dual task walking (p<0.01). Furthermore, the dual task cost-speed during cognitive dual task walking (30.09±16.91%) is higher than during motor dual task walking (13.19±7.29%; p<0.001). Therefore, the cognitive dual task gait seems to be more challenging to our participants. Although the exact reasons for improving motor dual task walking and single walking by cognitive dual task gait training is not immediately known, the difficulties or complexity of cognitive dual task gait training in this study is suggested. On the other hand, the cognitive function may be improved by our cognitive dual task gait training to result in better walking performances, since cognitive function is necessary for gait performance especially during dual tasking according to the capacity sharing theory [22–24]. However, we did not assess the changes of cognitive function after training in this study, and this possible explanation needs to be validated.

The gait variability, stride-to-stride fluctuations, is a marker for gait rhythmicity and automaticity, and increasing gait variability such as stride time variability is related to gait unsteadiness and fall risks [4]. In people with PD, alterations in walking include increased stride time variability, and dual tasking further increases this variability. It is interesting to note that the stride time variability during motor dual task walking was improved only after the motor dual task gait training, and such improvement was significant as compared with cognitive dual task gait training and general gait training. Similarly, the freezing of gait, as indicated by the results of the FOGQ, was also improved only after the motor dual task gait training. FOG is a debilitating symptom in patients with PD; however, the underlying mechanisms have not been fully elucidated [25]. Researchers suggested that step scaling is associated with FOG. It is known that the supplementary motor area (SMA) is responsible for generating ‘internal cues’ which is a necessary step to creating a sequenced and scaled movement. We thus speculate that SMA may be more activated during motor dual task gait training to result in improvement in stride time variability and FOG than cognitive dual task gait training or regular gait training. However, whether different training protocol can activate different brain areas needs further study. On the other hand, it has been indicated that faster walking speed may decrease the gait variability [26, 27]. Due to not improving the gait speed, we thus suggest that the motor dual task gait training had a direct effect on gait variability in the present study.

It is noted that the dual task cost-speed did not change significantly after either the motor or cognitive dual task gait training. Therefore, the improvement in gait speed during dual tasking was not significant enough as compared with the single walking indicating the dual task walking, motor or cognitive, is still a challenging task for our participants even after 12 sessions of dual task gait training. However, we did demonstrate the dual task cost-speed can be improved by 12-session dual task gait training in people with stroke [9]. It infers that patients with PD may need more time to re-establish the ability of dual task walking, and this inference may extend to participants’ concerns about falling as indicated by the FES-I score in the present study.

### Study limitations

The small sample size and large variance are our study limitations, and thus the results should be considered as preliminary and largely descriptive. Although the positive training effect was observed with relatively large effect size (Cohen’s d > 0.6) of inter-group difference, a larger randomized controlled trial is still recommended to validate the reported benefits of different dual task training. Second, lacking of follow-up assessment after 12-session intervention limits inferring the long term or maintenance effect of treatment. Third, the cognitive dual task training containing 8 different tasks might more complicated than motor dual task training (4 different tasks) to result in better effects. In addition, as participants in the present study were aware of the treatment allocation, and the expectation of benefit may bias the results of our outcomes.

## Conclusions

Our preliminary results demonstrated that a 12-session of cognitive dual task gait training decreased double support time during cognitive dual task walking, and motor dual task gait training reduced gait variability during motor dual task walking in people with PD. In addition, the cognitive dual task gait training improved the speed, stride length, and double support time under motor dual task walking and single walking. Different training strategy can be adopted for possibly different training effects in people with PD. As for clinical practice, we recommend implementing cognitive and motor dual task gait training as part of PD rehabilitation for functional walking abilities.

## Supporting information

S1 Table Cognitive dual task gait training program. (DOCX)

S2 Table Motor dual task gait training program. (DOCX)

S3 Table General gait training program. (DOCX)

S1 File CONSORT checklist. (DOC)

S2 File IRB protocol—English. (DOCX)
