# Supplementary material for: Cognitive and motor dual task gait training exerted specific training effects on dual task gait performance in individuals with Parkinson’s disease: A randomized controlled pilot study
Source: PLoS One. 2019 Jun 20;14(6):e0218180. doi: 10.1371/journal.pone.0218180 (PMC6586283; doi:10.1371/journal.pone.0218180)
Supplement: S1 Table — (DOCX) [file pone.0218180.s001.docx]

S1 Table. Cognitive dual task gait training program

| Week | Session | Repeating words | Counting a  3-digit number forward | Counting a  3-digit number backward | Answering  simple question | Reciting  shopping list | Talking | Reciting a  short sentence backward | Singing |
| --- | --- | --- | --- | --- | --- | --- | --- | --- | --- |
| 1 | 1 | Walking forward  x 5 min | Walking forward x 5 min | Walking forward  x 5 min |  |  |  |  |  |
|  |  | Walking on  S-shaped route  x 5 min | Walking on S-shaped route  x 5 min | Walking on S-shaped route  x 5 min |  |  |  |  |  |
|  | 2 | Walking and obstacle crossing  x 5 min | Walking and obstacle crossing x 5 min | Walking and obstacle crossing x 5 min | Walking forward  x 5 min | Walking forward x 5 min | Walking forward  x 5 min |  |  |
|  | 3 | Tandem walking  x 5 min | Tandem walking x 5 min | Tandem walking x 5 min | Walking on S-shaped route  x 5 min | Walking on S-shaped route  x 5 min | Walking on S-shaped route  x 5 min |  |  |
| 2 | 4 | Backward walking x 5 min | Backward walking x 5 min | Backward walking x 5 min | Walking and obstacle crossing x 5 min | Walking and obstacle crossing x 5 min | Walking and obstacle crossing x 5 min |  |  |
|  | 5 | Backward walking x 5 min | Backward walking x 5 min | Backward walking x 5 min | Walking and obstacle crossing x 5 min | Walking and obstacle crossing x 5 min | Walking and obstacle crossing x 5 min |  |  |
|  | 6 |  |  |  | Tandem walking x 5 min | Tandem walking x 5 min | Tandem walking x 5 min |  |  |
|  |  |  |  |  | Backward walking x 5 min | Backward walking x 5 min | Backward walking x 5 min |  |  |

S1 Table (continued) Cognitive dual task gait training program

| Week | Session | Repeating words | Counting a  3-digit number forward | Counting a  3-digit number backward | Answering simple question | Reciting  shopping list | Talking | Reciting a  short sentence backward | Singing |
| --- | --- | --- | --- | --- | --- | --- | --- | --- | --- |
| 3 | 7 |  |  |  | Tandem walking x 5 min | Tandem walking  x 5 min | Tandem walking  x 5 min |  |  |
|  |  |  |  |  | Backward walking x 5 min | Backward walking x 5 min | Backward walking x 5 min |  |  |
|  | 8 |  |  |  |  |  | Backward walking x 10 min | Walking forward  x 10 min | Walking forward  x 10 min |
|  | 9 |  |  |  |  |  |  | Walking forward  x 10 min | Walking forward  x 10 min |
|  |  |  |  |  |  |  |  | Walking on S-shaped route  x 5 min | Walking on S-shaped route  x 5 min |
| 4 | 10 |  |  |  |  |  |  | Walking on S-shaped route  x 10 min | Walking on S-shaped route  x 10min |
|  |  |  |  |  |  |  |  | Walking and obstacle crossing  x 5 min | Walking and obstacle crossing  x 5 min |
|  | 11 |  |  |  |  |  |  | Walking and obstacle crossing  x 10 min | Walking and obstacle crossing  x 10 min |
|  |  |  |  |  |  |  |  | Tandem walking  x 5 min | Tandem walking  x 5 min |
|  | 12 |  |  |  |  |  |  | Tandem walking  x 10 min | Tandem walking  x 10 min |
|  |  |  |  |  |  |  |  | Backward walking x 5 min | Backward walking x 5 min |
