# Supplementary material for: Cognitive and motor dual task gait training exerted specific training effects on dual task gait performance in individuals with Parkinson’s disease: A randomized controlled pilot study
Source: PLoS One. 2019 Jun 20;14(6):e0218180. doi: 10.1371/journal.pone.0218180 (PMC6586283; doi:10.1371/journal.pone.0218180)
Supplement: S2 Table — (DOCX) [file pone.0218180.s002.docx]

S2 Table. Motor dual task gait training program

| Week | Session | Holding one ball | Bouncing basketball with both hands | Bouncing basketball with either hand | Bouncing basketball with one hand and holding another basketball with the other hand |
| --- | --- | --- | --- | --- | --- |
| 1 | 1 | Walking forward x 5 min | Walking forward x 10 min |  |  |
|  |  | Walking on S-shaped  route x 5 min | Walking on S-shaped  route x 10 min |  |  |
|  | 2 | Walking and obstacle  crossing x 5 min | Walking forward x 10 min |  |  |
|  |  | Tandem walking x 5 min | Walking on S-shaped  route x 10 min |  |  |
|  | 3 | Backward walking x 10 min | Walking and obstacle  crossing x 10 min |  |  |
|  |  |  | Tandem walking x 10 min |  |  |
| 2 | 4 |  | Walking and obstacle  crossing x 10 min | Walking forward x 10 min |  |
|  |  |  | Tandem walking x 10 min |  |  |
|  | 5 |  | Backward walking x 10 min | Walking forward x 10 min |  |
|  |  |  |  | Walking on S-shaped  route x 10 min |  |
|  | 6 |  | Backward walking x 10 min | Walking on S-shaped  route x 10 min |  |
|  |  |  |  | Walking and obstacle  crossing x 10 min |  |

S2 Table (continued) Motor dual task gait training program

| Week | Session | Holding one ball | Bouncing basketball with both hands | Bouncing basketball with either hand | Bouncing basketball with one hand and holding another basketball with the other hand |
| --- | --- | --- | --- | --- | --- |
| 3 | 7 |  | Backward walking x 10 min | Walking and obstacle  crossing x 10 min |  |
|  |  |  |  | Tandem walking x 10 min |  |
|  | 8 |  |  | Walking and obstacle  crossing x 10 min |  |
|  |  |  |  | Tandem walking x 10 min |  |
|  |  |  |  | Backward walking x 10 min |  |
|  | 9 |  |  | Tandem walking x 10 min | Walking forward x 10 min |
|  |  |  |  | Backward walking x 10 min |  |
| 4 | 10 |  |  | Backward walking x 10 min | Walking forward x 10 min |
|  |  |  |  |  | Walking on S-shaped  route x 10 min |
|  | 11 |  |  |  | Walking on S-shaped  route x 10 min |
|  |  |  |  |  | Walking and obstacle  crossing x 10 min |
|  |  |  |  |  | Tandem walking x 10 min |
|  | 12 |  |  |  | Walking and obstacle  crossing x 10 min |
|  |  |  |  |  | Tandem walking x 10 min |
|  |  |  |  |  | Backward walking x 10 min |
