# Supplementary material for: Cognitive and motor dual task gait training exerted specific training effects on dual task gait performance in individuals with Parkinson’s disease: A randomized controlled pilot study
Source: PLoS One. 2019 Jun 20;14(6):e0218180. doi: 10.1371/journal.pone.0218180 (PMC6586283; doi:10.1371/journal.pone.0218180)
Supplement: S3 Table — (DOCX) [file pone.0218180.s003.docx]

S3 Table. General gait training program

| Week | Walking forward | Walking on  S-shaped route | Walking and  obstacle crossing | Tandem walking | Backward walking |
| --- | --- | --- | --- | --- | --- |
| 1 | 5 min | 5 min | 5 min |  |  |
| 2 |  | 5 min | 5 min | 5 min |  |
| 3 |  |  | 5 min | 5 min | 5 min |
| 4 |  |  | 5 min | 5 min | 5 min |
