# Supplementary material for: Cognitive and motor dual task gait training exerted specific training effects on dual task gait performance in individuals with Parkinson’s disease: A randomized controlled pilot study
Source: PLoS One. 2019 Jun 20;14(6):e0218180. doi: 10.1371/journal.pone.0218180 (PMC6586283; doi:10.1371/journal.pone.0218180)
Supplement: S2 File — (DOCX) [file pone.0218180.s005.docx]

**Cognitive and motor dual task gait training exerted specific training effects on dual task gait performance in individuals with Parkinson’s disease: A randomized controlled pilot study**

**(IRB title: Effects of cognitive and motor dual task gait training on dual task walking performance in individuals with Parkinson’s disease: A randomized controlled trial)**

**Chapter 1 Introduction**

**Section 1 Background and Motivation**

Mobility in daily living requires walking while performing simultaneously cognitive or motor tasks (dual task). Patients with Parkinson’s disease (PD) demonstrate gait impairments which are even exacerbated during dual task walking. The cognitive dual task and motor dual task affect gait performance in patients with PD, including step length, gait speed, cadence and stride variability. Especially, the cognitive dual task increased the freezing episodes in patients with PD. Motor dual task gait training can improve the gait performance in stroke patient, but such effect in PD patients is not immediately known. On the other hand, cognitive dual task training has been proposed to improve gait and dual task gait performance in patients with PD, however, these results have not been validated by randomized controlled trials. Furthermore, whether cognitive and motor dual task training exert different effects are not established yet.

**Section 2 Purpose**

The purpose of this study is to investigate the motor and cognitive dual task gait training on dual task walking performance and to compare the different effects of motor and cognitive dual gait training in individuals with PD.

**Section 3 Hypothesis**

1. Both cognitive and motor dual task training will improve the gait performance during dual task walking.
2. Cognitive dual task training may exert the better improvement while executing cognitive dual task walking than motor dual task group and control group.
3. Motor dual task training may exert the better improvement while executing motor dual task walking than cognitive dual task group and control group.

**Section 4 Importance**

Executing dual task walking is a common task in daily living. Patients with PD often have difficulties in performing dual tasking; furthermore, it may cause falling and decrease the quality of life.

This study will investigate the effects of cognitive and motor dual task walking training on gait performance. This information can provide a new training strategy in rehabilitation of Parkinson’s disease.

**Chapter 2 Literature review**

**Section 1 Dual task**

Dual-tasking is a performance observed when people do two things simultaneously. Unlike single task, dual task condition requires larger attentional resources and induces higher brain activities. According to the types of a secondary task, dual task walking can be classified as motor or cognitive. Motor dual task walking refers to conducting motor tasks such as carrying objects or manipulating objects during walking; cognitive dual task walking implies conducting attentional tasks, including mental tracking, verbal fluency or conversational tasks or memory tasks during walking.

Previous studies demonstrated the gait performance deteriorated during dual task walking. In healthy adults, reduced gait speed and stride length, and increased cadence while executing cognitive dual task walking were observed. To explain the interference of performance in dual tasking, there are few theories have been developed, including bottleneck theory and capacity sharing theory. The bottleneck theory implies that parallel processing may be impossible for certain mental operation at the same time, therefore, one or both of tasks will be delayed or otherwise impaired. The capacity sharing theory posits that multiple tasks share a certain amount of processing capacity. When more capacities are allocated to one task, it leaves less for other tasks done at the same time.

**Section 2 Dual tasking**

Patients with PD often have difficulties in performing dual tasking which has been suggested as relating to the limited processing resources, poor executive function, and decreased movement automaticity. Previous studies established the negative effect of cognitive dual task walking on gait performance in PD, included decreased gait speed and stride length and increased stride-to-stride variability and frezzing of gait. While executing motor dual task, Heinzel et al. reported the gait speed decreased while walking with box-checking task compared to single walking. Furthermore, this motor dual task walking can be used to predict falling in people with PD.

On the other hand, the impaired basal ganglia would also cause motor impairment such as performing dual tasking. Basal ganglia is responsible to control automatic movement. While executing dual task, if an automatic movement is controlled by basal ganglia then people will allocate more attention to another unskilled task. Conversely, populations who suffered basal ganglia impaired (e.g. patients with PD) will perform the dual task deteriorated. In addition, Salit et al. indicated that both healthy elderly and patient with Parkinson’s disease execute dual tasking walking with gait speed and stride length decrease. However, the gait variability increased during dual task walking only noted in patients with PD.

**Section 3 Dual task training**

An important principle in motor learning is training specificity of task-specific exercises to improve task performance with repetitions. Accordingly, it has been suggested that dual task training might have greater effects for improving dual task performance as compared to single task training. Subramaniam et al. and Lee et al. found significant improvements on balance ability and cognitive-motor interference under dual task conditions after combined balance and cognitive training. Regarding to improving dual task walking, Yang et al, found the gait speed, cadence, stride time, and stride length were improved during motor dual task walking (tray-carrying task while walking) after 4 weeks of motor dual task gait training in stroke. Plummer et al. also reported improvement in cognitive dual task gait speed after 12 sessions of gait training with additional cognitive task (cognitive dual task gait training) in stroke. Regarding to patients with PD, Collen et al and Sandra et al found the gait speed, cadence and stride length increased after single session cognitive dual task gait training. Galie et al, reported gait speed and variability improved after 4 weeks cognitive dual task training and the positive effects sustained after 1 month.

**Section 4 Summary**

Previous study pointed out the importance of dual tasking in daily living. Gait performance declined and fall risk elevated during dual task walking in patients with stroke.

There are few articles reported positive effects of dual task gait training in stroke. However, there is no article investigates the difference types of dual task training on different type of dual task walking in patients with Parkinson’s disease.

Therefore, this study is going to investigate the effects of cognitive and motor dual task gait training on gait performance in patients with Parkinson’s disease.

**Chapter 3 Research method**

**Section 1 Study protocol**

This is a randomized controlled trial. Thirty-six participants with Parkinson’s disease will be recruited in this study. An individual who was not involved with the study selected sealed envelopes to assign participants to one of the three treatment groups: cognitive dual task gait training (CDTT), motor dual task gait training (MDTT), or conventional physical therapy (CPT). All training sessions were 30 minutes long, and every training was administered three sessions per week for a total of four weeks by the same physical therapist. All outcomes were measured on the day before the training intervention began (pre) and on the day after the intervention was completed (post). (Figure 1)

**Section 2 Participants**

1. Inclusion criteria
2. Diagnosed idiopathic Parkinson’s disease
3. Hoehn and Yahr stage between 1 and 3
4. Stable medical condition
5. Ability to walk 10 meters independently without an assistive device
6. MMSE score ≧ 24
7. Exclusion criteria
8. Subjects were excluded if they had any other neurologic or orthopedic diseases that might interfere with participating in the study.

Screening

Recruiting participants through

inclusion criteria

Randomization (N=36)

CDTT group

(N=12)

MDTT group

(N=12)

p

CPT group

(N=12)

Pre-training

assessment

4 weeks intervention

Post-training

assessment

Figure 1. Flow chart

**Section 3 Intervention**

Participants in the CPT group received conventional gait training, which included walking forward, walking backward, walking on an S-shaped route, and obstacles crossing walking.

Participants in the CDTT group were instructed to perform cognitive tasks during diverse walking conditions on a level surface. The cognitive dual tasks included: (1) walking while repeating words, (2) walking while counting a 3-digit number forward, (3) walking while counting a 3-digit number backward, (4) walking while answering simple question “yes” or “no”, (5) walking while reciting a shopping list, (6) walking while talking, (7) walking while reciting a short sentence backward, and (8) walking while singing. Practices for these walking conditions included walking forward, walking on an S-shaped route, walking and obstacle crossing, tandem walking, and walking backward. Participants were challenged with increasingly difficult tasks as the training progressed.

Participants in the MDTT group were instructed to perform motor tasks during diverse walking conditions on a level surface. The motor dual tasks included: (1) walking while holding one ball (diameter = 20 cm) with both hands; (2) walking while bouncing a basketball (diameter = 24.6 cm) with both hands; (3) walking while bouncing a basketball (diameter = 24.6 cm) with either hand; and (4) walking while bouncing one basketball (diameter = 24.6 cm) with one hand and concurrently holding another basketball with the other hand (diameter = 20 cm). Practices for these walking conditions included walking forward, walking on an S-shaped route, walking and obstacle crossing, tandem walking, and walking backward. Participants were challenged with increasingly difficult tasks as the training progressed.

**Section 3 Outcome measure**

*Primary outcome*

1. **Dual task walking performance**

The gait performance was recorded and analyzed using the GAITRite walkway system (CIR System, Inc., Havertown, PA, USA) containing sensor pads connected to a laptop computer. This walkway is 4.75 m long and 0.89 m wide, with a 3.66-m long and 0.61-m wide pressure-sensitive area. When the participant walked along the walkway, the contact time and location of each footfall were recorded and analyzed using the application software.

In this study, participants will execute walking while performing cognitive task (WCT). Participants walk while subtracting 3 from an initial three-digit number serially and speaking out each calculated number as quickly as possible (e.g. 100, 97, 94). During walking while performing motor task (WMT), participants will walk while carrying a tray with a cup of water on the tray with their both hands without cup falling.

The gait parameters included speed (cm/s), cadence (steps/min), stride time (s), and stride length (cm) and double support time (s). In addition, the temporal gait variability (coefficient of variation = standard deviation/mean x 100%) of the stride time.

1. **Dual task effect**

Dual task effect will be calculated by dual task cost of speed (DTC-speed), the formula is shown below:

Dual task cost of speed $=\left| \frac{dual task speed-single task speed}{single task speed} \right|*100\%$

*Secondary outcome*

1. **Gait performance**

Participants execute single task walking on the GAITRite walkway system (CIR System, Inc., Havertown, PA, USA) wich recorded and analyzed gait performance. Gait parameters included speed (cm/s), cadence (steps/min), stride time (s), stride length (cm), double support time (s) and temporal gait variability.

1. **Cognitive function**

The executive control functions (ECF) will be assessed by C-EXIT 25 Chinese version which includes 25 items to test memory, speaking influent and sentence repeating. The intra-rater reliability was 0.7995, and the inter-rater reliability was 0.91.

1. **Risk of falling**

This study will use Fall Efficacy Scale (FES) to assess the confidence of falling of 10 items daily activities. This scale could predict the risk of falling and function decline in subjects. The test-retest reliability was 0.71.

1. **Quality of life**

The quality of life in a month will be assessed by Parkinson's Disease Questionnaire 39 (PDQ-39).

1. **Freezing of gait**
2. This study will use Freezing of Gait Questionnaire (FOGQ) which includes 6 items to assess the freezing gait. The intra-rater reliability was 0.94.
3. The number of freezing of gait

Using Liberty electromagnetic tracking system (Polhemus, Colchester, VT, USA) to record the number of freezing of gait during time up and go test (TUG). The data will be analysis through The Motion Monitor® system (TMM; Innovative Sport Training, Inc.,Chicago. IL. USA).

**Chapter 4 Data analysis**

All the data analysis will use SPSS 20.0. Descriptive statistics (mean ± standard deviation or frequency) were generated for all variables. Intergroup differences among baseline characteristics were evaluated using one-way ANOVA with repeated measure or χ2 analysis. Post hoc test will use Tukey method. Statistical significance was set as p < 0.05
